# What Influences People’s Tradeoff Decisions Between CO_2_ Emissions and Travel Time? An Experiment With Anchors and Normative Messages

**DOI:** 10.3389/fpsyg.2021.702398

**Published:** 2021-12-09

**Authors:** Hanna Andersson, Ulla Ahonen-Jonnarth, Mattias Holmgren, John E. Marsh, Marita Wallhagen, Fredrik Bökman

**Affiliations:** ^1^Department of Computer and Geospatial Sciences, University of Gävle, Gävle, Sweden; ^2^Department of Building Engineering, Energy Systems and Sustainability Science, University of Gävle, Gävle, Sweden; ^3^School of Psychology and Computer Science, University of Central Lancashire, Preston, United Kingdom; ^4^Engineering Psychology, Humans and Technology, Department of Business Administration, Technology and Social Sciences, Luleå University of Technology, Luleå, Sweden

**Keywords:** anchoring effect, normative message, travel time, tradeoff, environmental concern

## Abstract

One of the today’s greatest challenges is to adjust our behavior so that we can avoid a major climate disaster. To do so, we must make sacrifices for the sake of the environment. The study reported here investigates how anchors (extrinsic motivational-free information) and normative messages (extrinsic motivational information) influence people’s tradeoffs between travel time and carbon dioxide (CO_2_) emissions in the context of car travel and whether any interactions with environmental concern (an intrinsic motivational factor) can be observed. In this study, people received either a CO_2_, health or no normative message together with either a high anchor, a low anchor, or no anchor. People that received both a high anchor and a CO_2_ emission normative message were willing to travel for a longer time than those that only received a high anchor. If a low anchor was presented, no differences in willingness to travel for a longer time were found between the three different conditions of normative message groups, i.e., CO_2_ normative message, health normative message, or no normative message. People with higher concern for the environment were found to be willing to travel for a longer time than those with lower concern for the environment. Further, this effect was strongest when a high anchor was presented. These results suggest that anchors and normative messages are among the many factors that can influence people’s tradeoffs between CO_2_ emission and travel time, and that various factors may have to be combined to increase their influence over pro-environmental behavior and decisions.

## Introduction

Imagine that you have decided to travel from one city to another and have rented a petrol car for that purpose. When you arrive at the rental company, you receive the opportunity to change your petrol car to an equivalent electric car for the same cost. It will take a longer time to travel between the two cities in the electric car, but the CO_2_ emissions from the trip will be lower. How much more time would you be willing to let the journey take to reduce the CO_2_ emissions? This is an example of a tradeoff between what is good for the self (shorter travel time) and what is good for the environment (less CO_2_ emissions). Consumers frequently encounter tradeoffs between CO_2_ emissions and other aspects in our daily lives (e.g., when deciding between meals at a restaurant). When reflecting on these tradeoffs, it is necessary to consider how much we are willing to give up for our own sake to do something good for the environment, which is influenced by our own values and beliefs. Global warming is one of the greatest challenges today, and it is mainly due to human activities (Kramer et al., 2021). Even if we want to reduce our impact on the environment, psychological barriers stand in the way of behavior change (Gifford, 2011). In this paper, we explore how different types of information and motivational factors influence tradeoffs between travel time and CO_2_ emissions.

### Extrinsic Motivational Information

Informing people about others’ attitudes and behaviors is a strategy commonly used in campaigns to promote for example healthier, or pro-environmental behavior (Miller and Prentice, 2016). Seeing someone choose something healthy over something unhealthy may signal that choosing healthier options is more appropriate. Seeing someone reject something unhealthy in favor of something healthy may signal that choosing something unhealthy is inappropriate (Bergquist and Nilsson, 2019). How information is presented can be crucial in governing the effect it has on behavior change. For example, in a previous study promoting healthier behavior in the context of reducing salt intake a “loss frame,” i.e., cost of failing to engage in healthy behavior, was found to be more efficient than a “gain frame,” i.e., benefits of engaging in healthy behavior (Riet et al., 2010). An informational intervention (e.g., watching a movie with factual information and normative messages) can interact with pro-environmental values in influencing pro-environmental behavior. For example, watching the movie increased the participants’ knowledge about the environmental issues, but an increased intention to act pro-environmentally was only found for participants with strong biospheric values, i.e., with intrinsic motivational factors (Bolderdijk et al., 2013). Extrinsic motivational information in the form of a normative message has also been suggested to have an influence on a tradeoff decision between CO_2_ emissions and travel time (Bökman et al., 2021). In the present study, we are interested in investigating whether extrinsic motivational information in the form of a normative message that either is of relevance, or unrelated, to the situation influences people’s tradeoffs concerning time and CO_2_ emissions.

### Extrinsic Motivational-Free Information

Findings from research in psychology suggest that individual judgment and decision-making can be influenced by heuristics and biases (Tversky and Kahneman, 1974). Relevant to the current study is the so-called anchoring effect, which can be seen as motivational-free information, and which we use to study how extrinsic information influences tradeoffs. The anchoring effect is an extensively studied cognitive phenomenon, demonstrated in a variety of domains, such as general knowledge (Jacowitz and Kahneman, 1995), age estimation (Langeborg and Eriksson, 2016), payment (Jung et al., 2016), real estate evaluation (Northcraft and Neale, 1987), and estimation global warming (Joireman et al., 2010; for a review on the anchoring effect, see Furnham and Boo, 2011). In an anchoring task, participants first make a comparative judgment to a presented question, e.g., Did the Roman Emperor Julius Caesar weigh more or less than 70 pounds? (Wegener et al., 2001). In the following question, participants make an absolute judgment: How much do you think that the Roman Emperor Julius Caesar weighed? Another group receives the same questions, but with one difference. Instead of asking if they believe that the Roman Emperor Julius Caesar weighed more or less than 70 pounds^1^ (low anchor), they are asked if they believed that the emperor weighed more or less than 119 pounds^2^ (high anchor). People that receive a low anchor usually make a lower estimate than people that receive a high anchor – the anchoring effect.

Previous experiments have demonstrated that the anchoring effect is stronger for participants that are uncertain about the answer (Jacowitz and Kahneman, 1995) and that the effect is reduced when participants are asked to consider features that are inconsistent with the anchor (Chapman and Johnson, 1999). This indicates that when people make decisions, they automatically consider features consistent with the given information, but do not consider features that are inconsistent with such information, unless they are specifically asked to do so. In a study by Chapman and Johnson (1999), features were made more available through elaboration. The participants either listed things that they did that improved or threatened their health (elaboration on health condition), or things that they did that helped them avoid being a victim of a crime or made them more vulnerable to crime (crime elaboration condition). Later, the participants received both a question regarding crime and a question regarding health with or without anchors. The results showed that the participants in the health elaboration condition were more affected by the anchor in the health question and that participants in the crime elaboration condition were more affected by the anchor in the crime question. Responses from participants that did not receive any anchor fell in between responses from the two anchor conditions. Some studies have shown that having more knowledge reduces the anchoring effect (Wilson et al., 1996). However, there is a difference between having more knowledge and elaboration. According to Chapman and Johnson (1999), elaboration causes one to have more relevant information consistent with the judgment in mind, which might result in a stronger anchoring effect.

### Intrinsic Motivational Factors

Environmental concern, as a concept, has been widely used in environmental psychology research to reflect an overall attitude toward the environment (Fransson and Gärling, 1999). Based on value of Stern and Dietz (1994) basis theory for environmental attitudes, Schultz (2001) developed a measure of environmental concern that stems from the idea that people are concerned about adverse consequences of environmental problems for different reasons: for the biosphere, for other people, or for themselves. Some have suggested that these three measures are different from one another (Steg et al., 2005), but it has also been difficult to differentiate the altruistic value orientation from the biospheric value orientation (Hansla et al., 2008). Environmental concern has been demonstrated to be related to pro-environmental behaviors and intentions such as positive attitude toward green electricity (Hansla et al., 2008), higher willingness-to-pay for green products (Sörqvist et al., 2016), and renewable energy (Lin and Syrgabayeva, 2016). Higher concern for the environment has also been shown to correlate with stronger intentions to buy ecological products (Magnier and Schoormans, 2015), willingness to sacrifice spare time or money for the environment (Kuhlemeier et al., 1999), and it is also positively related to behavior intentions for mitigating climate change (Dienes, 2015). Crucially for the current study, people with higher environmental concern have also been found to be more affected by an anchor. Andersson et al. (2021) report that participants with higher environmental concern were willing to pay a higher price for an everyday food product when they received a high anchor, as well as a lower price when they received a low anchor, in comparison with people with lower environmental concern. Moreover, in a tradeoff task, Bökman et al. (2021) found that participants were willing to travel longer to decrease CO_2_ emissions when a normative message was combined with a high anchor, and that this interaction was strongest when environmental concern was high.

### Purpose

The results from a previous study investigating the tradeoff between travel time and CO_2_ emissions in the context of air travel in Sweden, with a university-based sample, showed that people were willing to travel for a longer time if they received both a high anchor and a normative message in comparison with people who received only a high anchor (Bökman et al., 2021). In the low anchor group, there was no difference between the two conditions (i.e., whether they received the normative message or not). In the current study, the aim was to further investigate normative messages as extrinsic motivational information, anchors as extrinsic motivational-free information, and environmental concern as intrinsic motivational factors when people make tradeoffs between travel time and CO_2_ emission. The sampling was undertaken on a large population and in a different country (England) to that of Bökman et al. (2021), and another vehicle (car) was used in the experiment. In addition, a normative message about health and questions without any anchor were included which were not used in the previous experiment by Bökman et al. (2021). Questions without an anchor were included as control conditions. The health normative message used in the present study was not directly related to the tradeoff question in contrast to the CO_2_ normative message, but of approximately the same length and holding a structurally similar content (a suggested maximum intake of salt or emission of CO_2_).

Given that anchors previously have been shown to influence willingness to travel for a longer time (Bökman et al., 2021), it is predicted that a high anchor will make participants willing to increase their travel time more in comparison with those that receive a low anchor (H1). The participants that receive no anchor are, on the other hand, predicted to be willing to travel for a longer time than those that receive a low anchor (H2a), but shorter time than those that receive a high anchor (H2b). A high anchor and a CO_2_ normative message are hypothesized to make people willing to travel for a longer time compared to those that receive a high anchor without any normative message (H3). Since the presence of a normative message has been shown to influence willingness to travel for a longer time (Bökman et al., 2021), it is predicted that a CO_2_ normative message will make participants willing to travel for a longer time than those that receive a health normative message (H4a) or no normative message (H4b). Considering that attitudes toward the environment have been shown to have an impact on pro-environmental intentions and behavior (e.g., Kuhlemeier et al., 1999; Steg et al., 2005; Bolderdijk et al., 2013; Dienes, 2015), it is hypothesized that people with higher concern for the environment will be willing to travel for a longer time (H5). Finally, people with high environmental concern are predicted to be more susceptible to the effects from a high anchor than their low concern counterparts (H6).

## Materials and Methods

### Participants

A sample of 1,076 participants living in England was recruited through the online crowd-sourcing platform Prolific Academic (61.5% women, mean age=36.5years, SD=11.7). A power analysis using G*Power 3.1.9.7 showed that a sample size of *n*=967 would be sufficient for the predictors in an ANOVA with nine groups with power set to 0.80, an alpha rate of 0.05, and effect sizes of *f*≥0.1 (as computed with G*Power, found under *F* test, ANOVA: fixed effects, special main effects, and interactions). One more power analysis was performed to analyze the interaction with nine groups with power set to 0.80, an alpha rate of 0.05, and effect sizes of *f*≥0.25 (as computed with G*Power, *F* test, ANOVA: fixed effects, special main effects, and interactions) which resulted in a suggested minimum sample size of 196 participants. The effect size *f*=0.1 for the main effect and *f*=0.25 for the interaction were determined from a similar previous study (see Bökman et al., 2021). As a sample size of 967 participants or higher was concluded to be appropriate according to the power calculation, a sample size of 1,076 was considered suitable due to the probability of some data loss following identification of ambiguous responses with screening procedures.

### Materials

An online service, Qualtrics, was used to construct the questionnaire. To measure the effects of an anchor and normative message when making a tradeoff, a three×three factorial design was used with three levels of anchor (no anchor, low anchor, and high anchor) and with three levels of normative message (no added normative message, CO_2_ normative message, and health normative message). The anchor values were selected after using a calibration group (*n*=90), that answered one of the three absolute judgment questions (with no added normative message, CO_2_ normative message, and health normative message). The calibration group was recruited from the same population through Prolific Academic. Participants selected for the calibration data collection were prevented from participating in the final study, using the “participate in a previous study” exclusion function in Prolific Academics. The high anchor value was set at the 85th percentile (8h and 30min) and the low anchor at the 15th percentile (5h and 30min) from the total distribution of the calibration group’s responses, in line with previous research (Jacowitz and Kahneman, 1995). The following is a description of how the comparison question and absolute judgment question used in the present experiment was presented to the low anchor group that received the CO_2_ normative message. For a detailed description, group by group, see Supplementary Material.

Assume that you have rented a petrol car to journey from Brighton to Manchester. The drive is estimated to take 5h and emit 61kg of carbon dioxide (CO_2_).

According to the Committee on Climate Change, a reduction to 4,500kg of CO_2_ emission per average United Kingdom household and year is required by 2030 to keep on track to achieve the United Kingdom-wide goal of reduction in CO_2_ emissions. This amounts to an average maximum of 36kg of CO_2_ per person and week.

If you got the opportunity to reduce the emissions to 20kg CO_2_ by renting an equivalent electric car at the same cost, would you be willing to let the journey take a longer time than 5h and 30min instead of 5h?

Yes/No.

If the participant selected “Yes,” they received this follow-up question:

(First, a repetition of the question you just answered)

* *the question and the normative message* *

(You answered “Yes” on the question above)

How much time would you be willing to let the journey take, at most, to reduce the emissions from 61kg of CO_2_ to 20kg CO_2_? Answer in hours and minutes.

If the participant answered “No” to the comparison question, the only difference was that it was stated that they answered “No” within the brackets, as they received the same information to follow and comparison question.

Participants filled in two mandatory fields for time: one for hours and one for minutes. The health normative message read: “According to the National Health Service, a reduction of 2.1g of salt a day is required to achieve the recommended daily consumption for adults to eat no more than 6g of salt a day, based on a recommendation from 2018. This amounts to a maximum of 42g per person and week.”

Used as an observational variable, participants answered the environmental concern questionnaire (Schultz, 2001). “How concerned are you that today’s environmental problems will affect…?” for 12 consequences on a nine-point scale ranging from 1 (not concerned) to 9 (very concerned). Finally, the participants answered if they hold a driver license or not (Yes/No).

### Design and Procedure

A between-participants design with the anchor (no anchor, low anchor, and high anchor) and with three levels of normative message (no added normative message, CO_2_ normative message, and health normative message) as independent variables and travel time as the dependent variable was used when collecting data. Participants were randomly allocated to one of the nine groups described above. After reading information about the study and answering “Yes” to the questions in the informed consent form, they started to answer the questions in the survey. The participants answered background questions regarding their age and gender, before answering the question concerning how long they would be willing to travel. After the travel question, participants answered the environmental concerns questionnaire before they were debriefed. The questionnaire took between 2 and 7min to complete.

To detect possible outliers, the interquartile range for the calibration group was calculated. Responses were considered outliers if their value exceeded the interquartile range of the calibration group times 2.4 (Hoaglin and Iglewicz, 1987). If a value was higher than the interquartile range times 2.4, winsorizing was used whereby the higher value was replaced by the highest accepted value of 11.10. A total of 19 participants were detected as outliers: no anchor and no normative message (three participants), no anchor and CO_2_ normative message (four participants), no anchor and health normative message (nine participants), high anchor and CO_2_ normative message (two participants) and finally high anchor and health normative message (one participant). No outliers were detected in the three low anchor groups or one of the high anchor groups (no normative message).

Schultz (2001) has distinguished between three types of environmental concern: biospheric (worried about consequences for nature and wildlife), altruistic (worried about humans including family and friends), and egoistic concern (worried about consequences for your lifestyle and health). In the present study, the correlation between the 12 items in environmental concern was high, *M*=6.8, SD=1.35, Cronbach’s *α*=0.92. As answers were highly correlated, all answers from the 12 questions were collapsed into one index instead of dividing them into three different environmental concern indexes. The participants were divided into three groups based on their mean score on all 12 questions in the environmental concern questionnaire. The groups were chosen to be as equal in size as possible and with the criterion that no one with the same score ended up in different groups. Participants in low environmental concern group one had a mean score ranging from 1.0 to 6.33. The medium environmental concern group had a score ranging from 6.42 to 7.33 and participants in the high environmental concern group three had a score ranging from 7.42 to 9.0 on the environmental concern questionnaire.

## Results

### Anchor and Normative Message

A three (no added normative message, CO_2_ normative message, and health normative message)×three (no, low or high anchor) univariate analysis of variance (ANOVA) was calculated. This revealed a main effect of anchor *F*(2, 1,067)=52.38, *p*<0.001, *η*^2^*_p_*=0.09, a main effect of normative message *F*(2, 1,067)=4.17, *p*=0.016, *η*^2^*_p_*=0.008, and an interaction between the anchor and normative message, *F*(4, 1,067)=3.08, *p*=0.015, *η*^2^*_p_*=0.011. When controlling for holding a driving license (yes vs. no) in a three×three ANCOVA, the results stay qualitatively the same [with a main effect of anchor *F*(2, 1,067)=52.52, *p*<0.001, *η*^2^*_p_*=0.09, a main effect of normative message *F*(2, 1,067)=4.1, *p*=0.017, *η*^2^*_p_*=0.008, and an interaction between the anchor and normative message, *F*(4, 1,067)=3.09, *p*=0.015, *η*^2^*_p_*=0.011, and no main effect of driving license *F*(1, 1,067)=0.822, *p*=0.365, *η*^2^*_p_*=0.001].

Single degree of freedom contrasts (Jaccard and Guilamo-Rames, 2002) were calculated using the methods of Wiens and Nilsson (2017), with standardized contrast weights (sum of absolute weights equals two) and a pooled error term to address the hypotheses that participants that received a high anchor would be willing to travel for a longer time in comparison with those that receive a low anchor (H1) or did not receive any anchor (H2b), that those who received no anchor would be willing to travel a longer time than those that did receive a low anchor (H2a), that a CO_2_ normative message would make participants willing to travel longer than those that received a health normative message (H4a) or no normative message (H4b), and that those that received a high anchor and CO_2_ normative message would be willing to travel for a longer time than those that only received a high anchor (H3).

**Figure 1:**
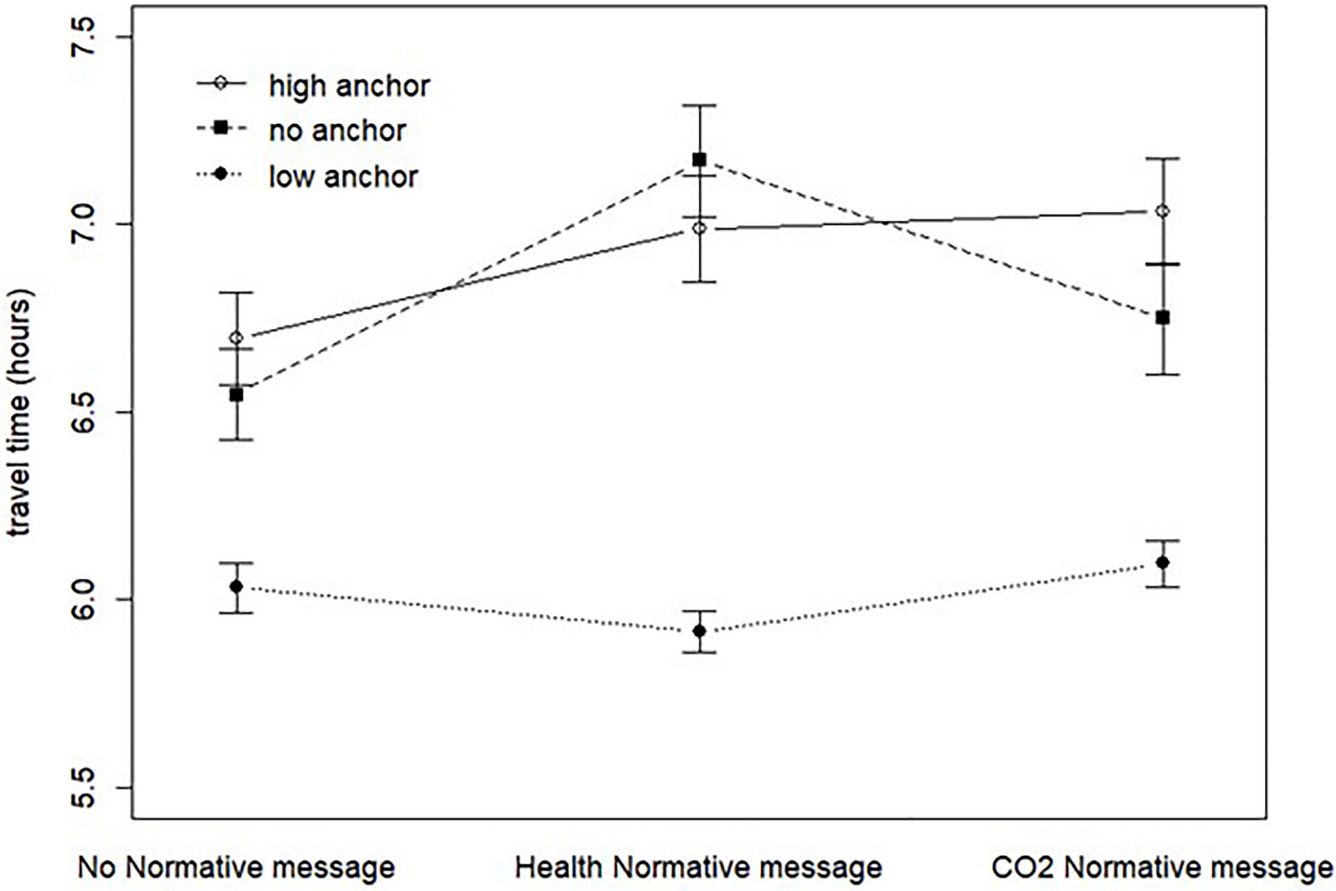
Judgments of travel time (willingness to travel) for anchor (no, low, or high anchor) and normative message (no information, health information, or CO_2_ information). Mean values with standard errors.

The contrast analysis (see Table 1) showed support for H1, so that participants that received a high anchor were willing to travel for a longer time than those receiving a low anchor. The contrast score of 0.89 is the difference between the means of the two conditions, a direct (unstandardized) effect size measure. On average, participants receiving a high anchor were willing to travel for 0.89h, or 53min, longer than those receiving a low anchor. The corresponding standardized effect score Cohen’s *d_c_* is 0.69 for H1. The contrast analysis also showed support for H2a, but not for H2b. A simple effect analysis (see Table 2) showed that the mean difference between the high and low anchor conditions (H1) was statistically significant for all three normative message conditions, as was the difference between no anchor and low anchor. The difference between the high anchor and no anchor conditions was not statistically significant at either of the three normative message conditions, with the CO_2_ normative message condition being closest (*p*=0.088). In summary, the main effect single degree of contrast analysis for the anchor factor suggests that the travel time answers with a high anchor and with no anchor do not differ, but that a low anchor significantly lowers the travel times, which is in line with what is shown in Figure 1. Support for H3 was found since those that received a high anchor and CO_2_ normative message were willing to travel for a longer time than those that only received a high anchor, see Table 1.

**Table 1:** Single degree of freedom contrasts for hypotheses H1–H4.^a^

	Score	95% CI	*t*	*p*	*d_c_*	
H1	0.89	[0.70, 1.08]	9.26	<0.001	0.69	High – low anchor
H2a	0.81	[0.62, 1.00]	8.41	<0.001	0.63	No – low anchor
H2b	0.08	[−0.10, 0.27]	0.87	0.19	0.06	High – no anchor
H3	0.34	[0.01, 0.66]	2.03	0.021	0.26	(High anchor, CO_2_ message) – (high anchor, no message)
H4a	−0.06	[−0.25, 0.12]	−0.66	0.75	−0.05	CO_2_ – health message
H4b	0.20	[0.01, 0.39]	2.11	0.018	0.16	CO_2_ – no message

a *When tests of statistical significance for the one-sided hypotheses were performed with Holms method for controlling for familywise type I errors the interpretation of the results did not change*.

**Table 2:** Simple main effect contrasts.^a^

	Score	95% CI	*t*	*p*	*d_c_*
*Simple contrasts for anchors*					
CO_2_ normative message					
High – low anchor	0.94	[0.61, 1.26]	5.63	<0.001	0.73
High – no anchor	0.28	[−0.04, 0.61]	1.71	0.088	0.22
No – low anchor	0.65	[0.33, 0.98]	3.92	<0.001	0.51
Health normative message					
High – low anchor	1.07	[0.74, 1.40]	6.42	<0.001	0.83
High – no anchor	−0.18	[−0.51, 0.15]	−1.09	0.28	−0.14
No – low anchor	1.25	[0.93, 1.58]	7.54	<0.001	0.98
No normative message					
High – low anchor	0.66	[0.34, 0.99]	3.99	<0.001	0.52
High – no anchor	0.15	[−0.18, 0.47]	0.90	0.37	0.12
No – low anchor	0.51	[0.19, 0.84]	3.10	0.002	0.40
*Simple contrasts for normative messages*					
High anchor					
CO_2_ – no message^*^					
CO_2_ – health message	0.05	[−0.28, 0.37]	0.28	0.78	0.04
Health – no message	0.29	[−0.04, 0.62]	1.75	0.081	0.23
No anchor					
CO_2_ – no message	0.20	[−0.12, 0.53]	1.23	0.22	0.16
CO_2_ – health message	−0.42	[−0.74, −0.09]	−2.52	0.012	−0.33
Health – no message	0.62	[0.30, 0.95]	3.76	<0.001	0.48
Low anchor					
CO_2_ – no message	0.06	[−0.26, 0.39]	0.39	0.70	0.05
CO_2_ – health message	0.18	[−0.15, 0.51]	1.09	0.27	0.14
Health – no message	−0.12	[−0.44,0.21]	−0.71	0.48	−0.09

* *See H3 in Table 1*.

a *Holms method for controlling for familywise type I errors did not change the interpretation of the results*.

### Environmental Concern and Travel Time

To address the hypothesis that participants with higher concern for the environment would be willing to travel for a longer time (H5), a linear regression analysis was performed to assess whether environmental concern predicted willingness to travel. The results showed a significant model [*F*(1, 1,074)=44.64, *p*<0.001] with an *R*^2^ of 0.040. Therefore, environmental concern explained 4% of the variation in travel time. Participants’ predicted willingness to travel is equal to 5.22+0.201 (EC) whereby willingness to travel is measured in hours. Participants’ willingness to travel increased 12min for each step of higher environmental concern.

### Anchor and Environmental Concern

**Figure 2:**
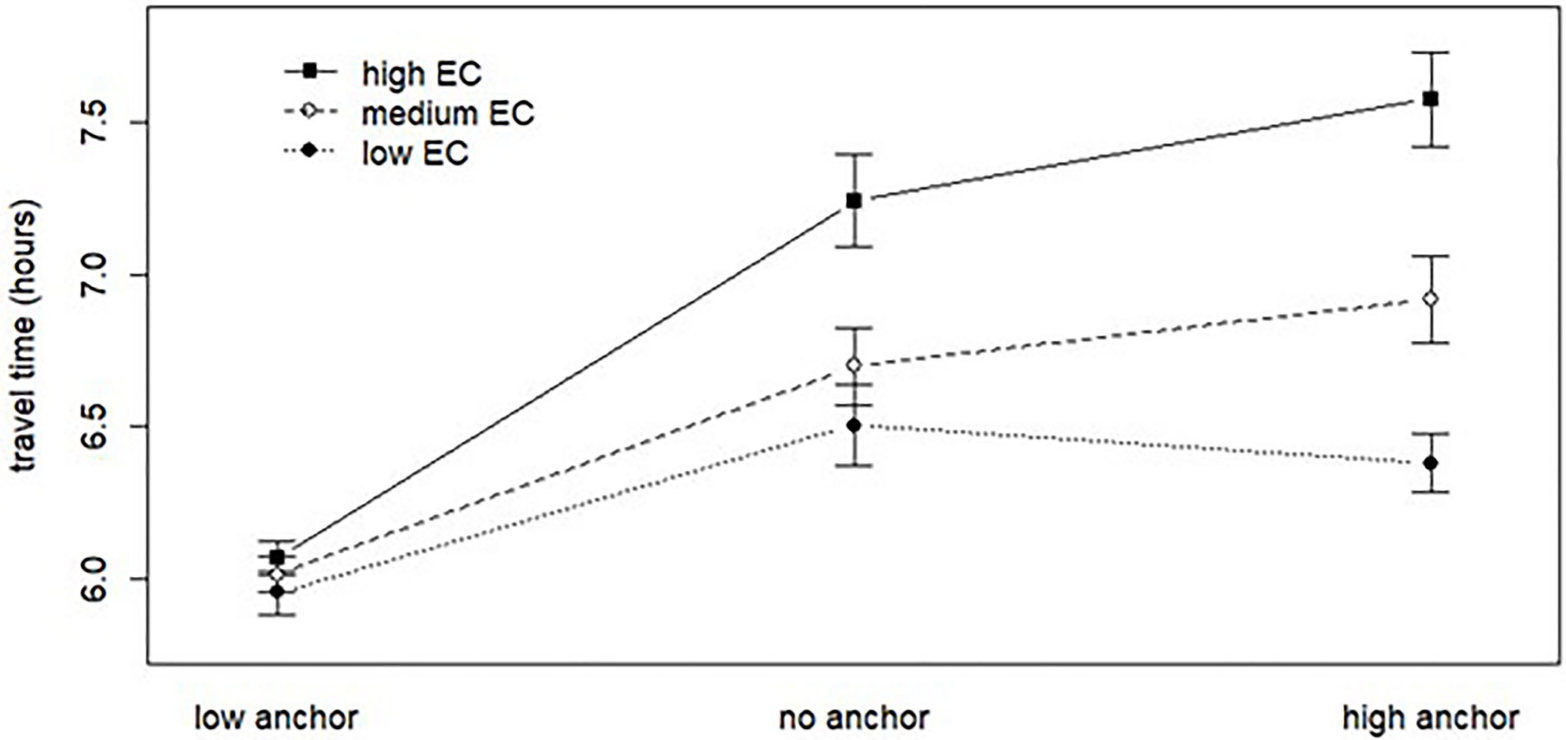
An illustration of the interaction between environmental concern (EC), divided in to three groups (low EC, medium EC, and high EC) and the three levels of anchor (low, high, or no anchor).

To address the hypothesis that people with high environmental concern were more susceptible to the effects of a high anchor than their low concern counterparts (H6), a three (no added normative message, CO_2_ normative message, and health normative message)×three (no, low or high anchor)×three (low, medium, and high environmental concern) ANOVA was calculated, wherein the participants have been divided into three environmental concern groups of approximately the same size, see design and procedure in the method section. There was a statistically significant interaction between anchor and environmental concern [*F*(4, 1,049)=6.55, *p*<0.001] and a main effect of environmental concern [*F*(2, 1,049)=27.52, *p*<0.001]. The analysis also revealed a main effect of anchor and normative message and an interaction between anchor and normative message already shown in the three×three ANOVA (see section “Anchor and Normative Message”). The interaction between environmental concern and anchor is visualized in Figure 2. Participants with higher environmental concern (group 3, EC 7.42–9.0) appear to be much more strongly affected by a high anchor than participants with low environmental concern (group 1, EC 1.0–6.33).

The interaction between environmental concern and anchor was further analyzed by means of a multiple regression (Table 3) of travel time answers on anchor and environmental concern, including interaction terms. The anchor factor with three levels was treated as two dummy categorical variables in the multiple regression, contrasting a low and a high anchor with the no anchor condition, while the mean-centered environmental concern was a continuous variable in the regression. The outcome of the multiple regression demonstrates a statistically significant interaction between environmental concern and both the anchor variables, going in the expected directions – that people with high concern for the environment were willing to travel for a longer time when they received a high anchor. Simple slope analysis shows significant positive slopes for EC both with a high anchor [*b*=0.39, 95% CI =(0.29, 0.48)] and with no anchor [*b*=0.21, 95% CI=(0.11, 0.30)], but not in the low anchor condition. Pairwise comparisons of the slopes show that the simple slope with a high anchor is significantly larger than with a low anchor (*p*<0.001) and with no anchor (*p*=0.018). An alternative spotlight analysis was made by looking at the simple effects of the anchors at three different values of environmental concern, *viz* at the mean of EC and one standard deviation below and above the mean. Both the high anchor and the no anchor conditions led to longer travel time answers than the low anchor condition for all three analysis points (*p*<0.001). However, it is only for the high EC point (mean+1 SD) that there is a significant simple effect of a high anchor relative to no anchor (*p*=0.004).

**Table 3:** Results from a multiple regression analysis of anchor and environmental concern on travel time answers, including interaction terms.

	*b*	*t*	95% CI
Intercept	6.82	103.68^***^	[6.69, 6.95]
Environmental concern	0.21	4.36^***^	[0.11, 0.30]
Low anchor	−0.81	−8.69^***^	[−1.00, −0.63]
High anchor	0.14	1.54	[−0.04, 0.33]
Environmental concern×Low anchor	−0.13	−1.98^*^	[−0.27, 0.00]
Environmental concern×High anchor	0.18	2.63^**^	[0.05, 0.31]

*Estimated unstandardized coefficients b, *t*-values and 95% confidence intervals [R^2^=0.153, $R_{\mathrm{adj}}^{2}$=0.149, *F*(5, 1,070)=38.71, p<0.001]*.

* *p<0.05*;

** *p<0.01 and*

*** *p<0.001*.

It was hypothesized that participants with higher environmental concern were going to be more susceptible to the high anchor than their low concern counterparts (H6). This hypothesis was supported, as participants with higher environmental concerns tended to be more influenced by a high anchor than their lower concern counterparts. Although participants who received a high anchor did not answer with significantly longer travel times than participants who did not receive any anchor (See H2b, section “Anchor and Normative Message”), there was a significant interaction between high anchor and environmental concern, and the spotlight analysis showed that among the participants with highest environmental concern the travel time answers are higher in the high anchor than in the no anchor condition.

## Discussion

The results of the current study demonstrated that participants that received both a high anchor (extrinsic motivational-free information) and a CO_2_ normative message (extrinsic motivational information) were willing to travel for a longer time in comparison with those that received a high anchor without any normative message (support for H3). Further, the participants that received the CO_2_ normative message were willing to travel for a longer time in comparison with those that did not receive a normative message (support for H4b). However, participants who received the CO_2_ normative message were surprisingly not willing to travel longer than those who received the health normative message (no support for H4a). In fact, participants who received the health normative message were willing to travel longer than those who did not received any normative message. Thus, participants receiving some normative messages were willing to travel for a longer time than those that did not receive a normative message. It was also found that people that received a high anchor or no anchor were willing to travel for a longer time in comparison with those that received a low anchor (support for H1 and H2a). Notably there was no difference between the high anchor and no anchor groups, hence no support for H2b. This was somewhat surprising given the previous studies have found that the answer from the no anchor groups can fall in between the high and low anchor answers (see, e.g., Chapman and Johnson, 1999). The results revealed that people with higher concern for the environment were willing to travel for a longer time than the ones that were less concerned for the environment (support for H5), indicating that people with higher concern also are willing to make a larger sacrifice for the sake of the environment. Moreover, people with high environmental concern were more susceptible to the effects from high anchor than their low concern counterparts (support for H6).

The results are similar to the one found in the study by Bökman et al. (2021), wherein people were found to be willing to travel for a longer time, in the context of air travel in Sweden, when they received both a high anchor and CO_2_ normative message in comparison with only a high anchor. Further, Wu and Cheng (2011) found that a positive attribute together with a high anchor induces higher willingness to pay responses than other combinations (i.e., information framed in positive or negative terms with high or low anchor present). In the present study, it was hypothesized that English people that received both a high anchor and a CO_2_ normative message would be willing to travel for a longer time than those that received only a high anchor (H3), and support for the hypothesis was found. On the contrary, in the low anchor condition, the three groups receiving different types of messages, i.e., CO_2_, health, or no normative message did not differ from each other. The results from the present study shed some light on the generalizability on how different types of external cues influence people when they make a tradeoff between travel time and CO_2_ emissions.

The current study, like many previous results, demonstrated the robustness of the anchoring effect. For example, previous research has found that even experts are susceptible to the anchoring effect (Northcraft and Neale, 1987; Vestlund et al., 2009). Wu and Cheng (2011) found that people with less knowledge about a target product were more susceptible to both anchoring and framing effects when stating their willingness to pay in an online shopping task. In our study, the information given to participants included a normative message that puts the CO_2_ emissions caused by the travel into context. Interestingly, if a low anchor was presented in this study, the normative message given did not affect participants’ willingness to travel for a longer time. The differences between the three normative message manipulations only manifest in the high and no anchor conditions.

Both extrinsic motivational information used, a CO_2_ normative message and a health normative message, seem to increase participants’ willingness to travel for a longer time in comparison with those that did not receive any normative message. Why did a normative health message also increase people’s willingness to travel? This was not expected and does not correspond with our hypothesis H4a. Both types of normative message were similar in such a way that they both sent a “do not message,” that has been shown to have a stronger effect on people’s choices in comparison with sending a “do message” (Bergquist and Nilsson, 2019). Chapman and Johnson (1999) showed that subjects who elaborated on a judgment domain (and thus had more information accessible) were more affected by the anchor in the domain wherein they had more accessible information. Providing a normative message with information on CO_2_ emissions can be characterized as a form of “issue framing” (Druckman, 2004), whereby the personal obligation to reduce CO_2_ emissions is emphasized. Participants that received one of the two normative messages had more information to consider when thinking about the tradeoff. This might have resulted in them generating more anchor-consistent target features which, according to Selective Accessibility Model (Mussweiler and Strack, 1999) and Anchoring as Activation (Chapman and Johnson, 1999), would result in a larger effect of the anchor. However, it is of relevance to point out that the health normative message used in the present study was not directly related to the tradeoff-question. On the other hand, many environmental issues are closely related to health aspects (e.g., eating eco-labeled groceries might both be an act done with the intention to reduce climate change or to eat more nutritious food to become healthier), and Chevance et al. (2021) suggest that there are bi-directional associations between health-related behaviors and climate change. Therefore, it is possible that reading a normative message about health might have made people more willing to make travel judgements in a pro-environmental way.

Environmental concern, the intrinsic motivational factor studied, influenced people’s willingness to sacrifice their time to reduce CO_2_ emissions. This finding, that people that are more concerned for the environment were willing to travel for a longer time, is in line with previous research (Bökman et al., 2021). Similarly, previous studies have shown that high environmental concern is related to pro-environmental behavior, e.g., willingness to reduce household energy consumption (Steg et al., 2005). People with positive attitudes toward the nature and the environment have also been found to be willing to pay higher taxes, higher prices on products and services (Joireman et al., 2010) as well as willing to sacrifice spare time or money for the environment (Kuhlemeier et al., 1999). Further, Huffman et al. (2014) studied recycling and found a significant interaction between anthropocentrism (e.g., self-containment from nature) and recycling attitudes. When individuals with strong recycling attitudes and low anthropocentrism orientation (in comparison with a high anthropocentrism orientation) were more likely to recycle. But participants with a weak recycling attitude were more likely to present observed recycling behavior if they had high anthropocentrism compared to those with low anthropocentrism. Previous research has also suggested that an informational intervention can make those that strongly care about the environment more prone to act in a pro-environmental way (Bolderdijk et al., 2013). In this line of thought, it is not surprising that people with high environmental concern are more susceptible to this experimental push compared to people with low concern for the environment, especially when they receive a high anchor. However, the analysis of environmental concern was not included in our power analysis so this analysis might be underpowered.

A limitation of the present study is that the scenario in which the participants answered the question is hypothetical, as none of the participants are at the car-rental receiving this option to switch from a petrol car to an electric car. Kormos and Gifford (2014) have pointed out that self-reported behavior does not always translate to actual behavior. Self-reported recycling behavior has for example been found to correlate, but not strongly, with observed recycling behavior (Huffman et al., 2014). Although it is possible to present participants with a pro-environmental behavior task with real consequences within a laboratory (Lange et al., 2018), in the current study the scenario used is straightforward and could possibly be a scenario in which some people might find themselves in a not-too-distant future. The same scenario could be of interest in future research.

In the current study, there might also be other confounders such as how car-dependent people are in their daily life, and opinions of electric cars or cars in general. Some participants might find longer car-travel unthinkable while others are used to it. In the present study we controlled for having a driving license, demonstrated to have no impact of the main outcomes of the study. Future research should consider including more covariates of relevance for these types of scenarios.

The results indicate that tradeoffs between the self and the environment can be influenced by external cues. Individual differences are important to consider, and people with a high concern for the environment seem to be more susceptible to the effects of a high anchor. From an applied perspective, it was interesting to find that, compared to the condition with no anchor, the low anchor appears to have the effect of pushing judgements down rather than the high anchor pushing the judgements up. In this tradeoff question, the low anchor could be of moral relevance. People that want to act in a pro-environmental way, might think that it is enough to answer a little higher than the low anchor. But when no anchor was present, people were willing to travel for a longer time to reduce the CO_2_ emissions. As living car-free is one of the biggest efforts, we can make to combat climate change (Wynes and Nicholas, 2017), it is of great importance, for future studies, to investigate tradeoffs between travel time and CO_2_ emissions in real case scenarios.

## Data Availability Statement

The raw data supporting the conclusions of this article will be made available by the authors, without undue reservation.

## Ethics Statement

Ethical review and approval was not required for the study on human participants in accordance with the local legislation and institutional requirements. The patients/participants provided their written informed consent to participate in this study.

## Author Contributions

HA: conceptualization, methodology, investigation, formal analysis, visualization, writing – original draft, and writing – review and editing. UA-J: conceptualization, methodology, writing – review and editing, and supervision. FB: conceptualization, methodology, formal analysis, visualization, writing – review and editing, and supervision. MH: formal analysis and writing – review and editing. JM: conceptualization and writing – review and editing. MW: writing – review and editing and supervision. All authors contributed to the article and approved the submitted version.

## Conflict of Interest

The authors declare that the research was conducted in the absence of any commercial or financial relationships that could be construed as a potential conflict of interest.

## Publisher’s Note

All claims expressed in this article are solely those of the authors and do not necessarily represent those of their affiliated organizations, or those of the publisher, the editors and the reviewers. Any product that may be evaluated in this article, or claim that may be made by its manufacturer, is not guaranteed or endorsed by the publisher.

## Footnotes

^1^32 kg.

^2^54 kg.
