# Supplementary material for: What Influences People’s Tradeoff Decisions Between CO2 Emissions and Travel Time? An Experiment With Anchors and Normative Messages
Source: Front Psychol. 2021 Dec 9;12:702398. doi: 10.3389/fpsyg.2021.702398 (PMC8699112; doi:10.3389/fpsyg.2021.702398)
Supplement: Supplementary file 1 [file Data_Sheet_1.PDF]

Supplementary Material

Here is a breakdown of the questions received by the nine groups in the study.

Groups D-I received an anchor and therefore two questions. First, a Yes/No question followed by the absolute judgment question. The two questions were presented on two different pages in the digital survey. The two pages are here illustrated by a horizontal line. Group A-C did not receive an anchor and therefore only an absolute judgment question. Note that the labeling of groups is only used in the Supplementary Material for clarification. In the article, the groups are referred to what type of question they received (e.g., a low anchor and health normative message).

	No anchor	Low anchor	High anchor
No normative message	A	D	G
Health normative message	B	E	H
CO_2 normative message	C	F	I

Group A

Assume that you have rented a petrol car to journey from Brighton to Manchester. The drive is estimated to take 5 hours and emit 61 kg of carbon dioxide (CO_2).

How much time would you be willing to let the journey take, at most, to reduce the emissions from 61 kg of CO_2 to 20 kg CO_2? Answer in hours and minutes.

Group B

Assume that you have rented a petrol car to journey from Brighton to Manchester. The drive is estimated to take 5 hours and emit 61 kg of carbon dioxide (CO_2).

According to the National Health Service, a reduction of 2.1g of salt a day is required to achieve the recommended daily consumption for adults to eat no more than 6g of salt a day, based on a recommendation from 2018. This amounts to a maximum of 42g per person and week.

How much time would you be willing to let the journey take, at most, to reduce the emissions from 61 kg of CO_2 to 20 kg CO_2? Answer in hours and minutes.

Group C

Assume that you have rented a petrol car to journey from Brighton to Manchester. The drive is estimated to take 5 hours and emit 61 kg of carbon dioxide (CO_2).

According to the Committee on Climate Change, a reduction to 4 500 kg of CO_2 emission per average UK household and year is required by 2030 to keep on track to achieve the UK-wide goal of reduction in CO_2 emissions. This amounts to an average maximum of 36 kg of CO_2 per person and week.

How much time would you be willing to let the journey take, at most, to reduce the emissions from 61 kg of CO_2 to 20 kg CO_2? Answer in hours and minutes.

Group D

Assume that you have rented a petrol car to journey from Brighton to Manchester. The drive is estimated to take 5 hours and emit 61 kg of carbon dioxide (CO_2).

If you got the opportunity to reduce the emissions to 20 kg CO_2 by renting an equivalent electric car at the same cost, would you be willing to let the journey take a longer time than 5 hours and 30 minutes instead of 5 hours?

Yes

No

(First, a repetition of the question you just answered)

“Assume that you have rented a petrol car to journey from Brighton to Manchester. The drive is estimated to take 5 hours and emit 61 kg of carbon dioxide (CO_2).

If you got the opportunity to reduce the emissions to 20 kg CO_2 by renting an equivalent electric car at the same cost, would you be willing to let the journey take a longer time than 5 hours and 30 minutes instead of 5 hours?”

(You answered "Yes (No)" [1]on the question above)

How much time would you be willing to let the journey take, at most, to reduce the emissions from 61 kg of CO_2 to 20 kg CO_2? Answer in hours and minutes.

[1] Only a ”Yes” or a ”No” was stated here depending on participants answer to the previous question.

Group E

Assume that you have rented a petrol car to journey from Brighton to Manchester. The drive is estimated to take 5 hours and emit 61 kg of carbon dioxide (CO_2).

According to the National Health Service, a reduction of 2.1g of salt a day is required to achieve the recommended daily consumption for adults to eat no more than 6g of salt a day, based on a recommendation from 2018. This amounts to a maximum of 42g per person and week.

If you got the opportunity to reduce the emissions to 20 kg CO_2 by renting an equivalent electric car at the same cost, would you be willing to let the journey take a longer time than 5 hours and 30 minutes instead of 5 hours?

Yes

No

(First, a repetition of the question you just answered)

“Assume that you have rented a petrol car to journey from Brighton to Manchester. The drive is estimated to take 5 hours and emit 61 kg of carbon dioxide (CO_2).

According to the National Health Service, a reduction of 2.1g of salt a day is required to achieve the recommended daily consumption for adults to eat no more than 6g of salt a day, based on a recommendation from 2018. This amounts to a maximum of 42g per person and week.

If you got the opportunity to reduce the emissions to 20 kg CO_2 by renting an equivalent electric car at the same cost, would you be willing to let the journey take a longer time than 5 hours and 30 minutes instead of 5 hours?”

(You answered "Yes (No)" on the question above)

How much time would you be willing to let the journey take, at most, to reduce the emissions from 61 kg of CO_2 to 20 kg CO_2? Answer in hours and minutes.

Group F

Assume that you have rented a petrol car to journey from Brighton to Manchester. The drive is estimated to take 5 hours and emit 61 kg of carbon dioxide (CO_2).

According to the Committee on Climate Change, a reduction to 4 500 kg of CO_2 emission per average UK household and year is required by 2030 to keep on track to achieve the UK-wide goal of reduction in CO_2 emissions. This amounts to an average maximum of 36 kg of CO_2 per person and week.

If you got the opportunity to reduce the emissions to 20 kg CO_2 by renting an equivalent electric car at the same cost, would you be willing to let the journey take a longer time than 5 hours and 30 minutes instead of 5 hours?

Yes

No

(First, a repetition of the question you just answered)

“Assume that you have rented a petrol car to journey from Brighton to Manchester. The drive is estimated to take 5 hours and emit 61 kg of carbon dioxide (CO_2).

According to the Committee on Climate Change, a reduction to 4 500 kg of CO_2 emission per average UK household and year is required by 2030 to keep on track to achieve the UK-wide goal of reduction in CO_2 emissions. This amounts to an average maximum of 36 kg of CO_2 per person and week.

If you got the opportunity to reduce the emissions to 20 kg CO_2 by renting an equivalent electric car at the same cost, would you be willing to let the journey take a longer time than 5 hours and 30 minutes instead of 5 hours?”

(You answered "Yes (No)" on the question above)

How much time would you be willing to let the journey take, at most, to reduce the emissions from 61 kg of CO_2 to 20 kg CO_2? Answer in hours and minutes.

Group G

Assume that you have rented a petrol car to journey from Brighton to Manchester. The drive is estimated to take 5 hours and emit 61 kg of carbon dioxide (CO_2).

If you got the opportunity to reduce the emissions to 20 kg CO_2 by renting an equivalent electric car at the same cost, would you be willing to let the journey take a longer time than 8 hours and 30 minutes instead of 5 hours?

Yes

No

(First, a repetition of the question you just answered)

“Assume that you have rented a petrol car to journey from Brighton to Manchester. The drive is estimated to take 5 hours and emit 61 kg of carbon dioxide (CO_2).

If you got the opportunity to reduce the emissions to 20 kg CO_2 by renting an equivalent electric car at the same cost, would you be willing to let the journey take a longer time than 8 hours and 30 minutes instead of 5 hours?”

(You answered "Yes (No)" on the question above)

How much time would you be willing to let the journey take, at most, to reduce the emissions from 61 kg of CO_2 to 20 kg CO_2? Answer in hours and minutes.

Group H

Assume that you have rented a petrol car to journey from Brighton to Manchester. The drive is estimated to take 5 hours and emit 61 kg of carbon dioxide (CO_2).

According to the National Health Service, a reduction of 2.1g of salt a day is required to achieve the recommended daily consumption for adults to eat no more than 6g of salt a day, based on a recommendation from 2018. This amounts to a maximum of 42g per person and week.

If you got the opportunity to reduce the emissions to 20 kg CO_2 by renting an equivalent electric car at the same cost, would you be willing to let the journey take a longer time than 8 hours and 30 minutes instead of 5 hours?

Yes

No

(First, a repetition of the question you just answered)

“Assume that you have rented a petrol car to journey from Brighton to Manchester. The drive is estimated to take 5 hours and emit 61 kg of carbon dioxide (CO_2).

According to the National Health Service, a reduction of 2.1g of salt a day is required to achieve the recommended daily consumption for adults to eat no more than 6g of salt a day, based on a recommendation from 2018. This amounts to a maximum of 42g per person and week.

If you got the opportunity to reduce the emissions to 20 kg CO_2 by renting an equivalent electric car at the same cost, would you be willing to let the journey take a longer time than 8 hours and 30 minutes instead of 5 hours?”

(You answered "Yes (No)" on the question above)

How much time would you be willing to let the journey take, at most, to reduce the emissions from 61 kg of CO_2 to 20 kg CO_2? Answer in hours and minutes.

Group I

Assume that you have rented a petrol car to journey from Brighton to Manchester. The drive is estimated to take 5 hours and emit 61 kg of carbon dioxide (CO_2).

According to the Committee on Climate Change, a reduction to 4 500 kg of CO_2 emission per average UK household and year is required by 2030 to keep on track to achieve the UK-wide goal of reduction in CO_2 emissions. This amounts to an average maximum of 36 kg of CO_2 per person and week.

If you got the opportunity to reduce the emissions to 20 kg CO_2 by renting an equivalent electric car at the same cost, would you be willing to let the journey take a longer time than 8 hours and 30 minutes instead of 5 hours?

Yes

No

(First, a repetition of the question you just answered)

“Assume that you have rented a petrol car to journey from Brighton to Manchester. The drive is estimated to take 5 hours and emit 61 kg of carbon dioxide (CO_2).

According to the Committee on Climate Change, a reduction to 4 500 kg of CO_2 emission per average UK household and year is required by 2030 to keep on track to achieve the UK-wide goal of reduction in CO_2 emissions. This amounts to an average maximum of 36 kg of CO_2 per person and week.

If you got the opportunity to reduce the emissions to 20 kg CO_2 by renting an equivalent electric car at the same cost, would you be willing to let the journey take a longer time than 8 hours and 30 minutes instead of 5 hours?”

(You answered "Yes (No)" on the question above)

How much time would you be willing to let the journey take, at most, to reduce the emissions from 61 kg of CO_2 to 20 kg CO_2? Answer in hours and minutes.
